# MENSAdb: a thorough structural analysis of membrane protein dimers

**DOI:** 10.1093/database/baab013

**Published:** 2021-04-05

**Authors:** Pedro Matos-Filipe, António J Preto, Panagiotis I Koukos, Joana Mourão, Alexandre M J J Bonvin, Irina S Moreira

**Affiliations:** Center for Neuroscience and Cell Biology, University of Coimbra, Coimbra 3005-504, Portugal; Center for Neuroscience and Cell Biology, University of Coimbra, Coimbra 3005-504, Portugal; PhD Programme in Experimental Biology and Biomedicine, Institute for Interdisciplinary Research, University of Coimbra, Coimbra, 3030-789, Portugal; Bijvoet Centre for Biomolecular Research, Faculty of Science—Chemistry, Utrecht University, Utrecht, 3584, CH, Netherlands; Center for Neuroscience and Cell Biology, University of Coimbra, Coimbra 3005-504, Portugal; Bijvoet Centre for Biomolecular Research, Faculty of Science—Chemistry, Utrecht University, Utrecht, 3584, CH, Netherlands; Department of Life Sciences, University of Coimbra, Coimbra, 3000-456, Portugal; Center for Neuroscience and Cell Biology, Center for Innovative Biomedicine and Biotechnology, University of Coimbra, Coimbra, Portugal

## Abstract

Membrane proteins (MPs) are key players in a variety of different cellular processes and constitute the target of around 60% of all Food and Drug Administration–approved drugs. Despite their importance, there is still a massive lack of relevant structural, biochemical and mechanistic information mainly due to their localization within the lipid bilayer. To help fulfil this gap, we developed the MEmbrane protein dimer Novel Structure Analyser database (MENSAdb). This interactive web application summarizes the evolutionary and physicochemical properties of dimeric MPs to expand the available knowledge on the fundamental principles underlying their formation. Currently, MENSAdb contains features of 167 unique MPs (63% homo- and 37% heterodimers) and brings insights into the conservation of residues, accessible solvent area descriptors, average *B*-factors, intermolecular contacts at 2.5 Å and 4.0 Å distance cut-offs, hydrophobic contacts, hydrogen bonds, salt bridges, π–π stacking, T-stacking and cation–π interactions. The regular update and organization of all these data into a unique platform will allow a broad community of researchers to collect and analyse a large number of features efficiently, thus facilitating their use in the development of prediction models associated with MPs.

**Database URL**: http://www.moreiralab.com/resources/mensadb.

## Introduction

Membrane proteins (MPs) account for around 15–39% of the human proteome (1, 2). They assume a critical role in a vast set of cellular and physiological mechanisms, including molecular transport, nutrient uptake, toxin and waste product clearance, respiration and signalling (3). While roughly 60% of all Food and Drug Administration (FDA)–approved drugs target MPs, there is a shortage of structural and biochemical data about them mainly due to their localization in the lipid bilayer (4, 5). In the last years, a daunting challenge of drug discovery has been the development of compounds that can target the ‘undruggable’ regions of MPs, enabling the modulation of protein–lipid, protein–nucleic acid and protein–protein interactions (PPIs) (6, 7). In this respect, being able to characterize the structural and physicochemical properties of MPs as well as their interactions and interfaces is essential to develop improved and more targeted therapies as well as to discover new drug targets. Particular features of proteins, such as electrostatic interactions (8), hydrophobic effects (9) or ‘hot-spot’ residues (10–13), were shown to contribute to the affinity and specificity of PPIs. Other well-characterized properties of proteins are the evolutionary conservation and distribution of their amino acids. These two features contribute the most to the prediction of functionally essential residues, as highlighted by several publications (14–17). While many studies have dealt with soluble systems, there is a significant lack of in-depth analysis of MP complexes and their interactions.

**Figure 1.**
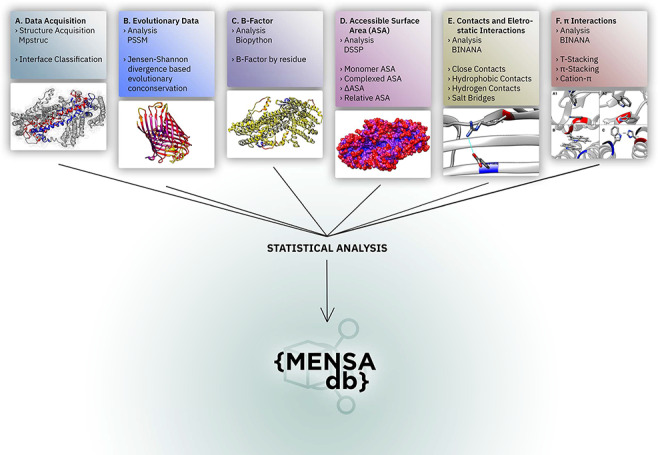
Overall representation of MENSAdb. Boxes A–F illustrate the steps involving the data collection, evolutionary conservation, *B*-factor, accessible surface and PPI analysis. Each box contains an example of the proteinic motifs under the scope of this work. (A) Interface between chains A and B of the STRA6 receptor for retinol uptake in *Danio rerio* (PDBid: 5SY1) (55). (B) Representation of evolutionary conservation of protein motifs (purple being more conserved and yellow less conserved) in the chain P of a hedgehog auto-processing domain in *Drosophila melanogaster* (PDBid: 1AT0) (56). (C) and (D) Average *B*-factor and complexed accessible surface area, respectively, of the chains A and B of 5SY1 (55). (E) Salt bridge between GLU120 and ARG161 of the chain Q of the sucrose-specific porin (PDBid: 1A0T) of *Salmonella*  *typhimurium* (57). (F) The spectrum of π systems predicted: (A1 and A2) T-stacking motif between TRP25 (chain L) and TRP255 (chain M) from *Rattus norvegicus* S100B protein (PDBid: 1XYD) (58) is represented from two perspectives; (B) illustration of a π–π stacking structure between TRP262 (chain A) and TRP262 (chain B) from *Archaeoglobus fulgidus* CDP-alcohol phosphotransferase (PDBid: 4O6M) (59) and (C) cation–π interaction between HIS275 (chain B) and TRP175 (chain C) from *Escherichia coli* formate dehydrogenase-N (PDNid: 1KQF) (60).

We present here the MEmbrane protein dimer Novel Structure Analyser database (MENSAdb), the first interactive web application exposing a comprehensive and thorough array of fundamental features of dimer surfaces of MPs and their interfacial regions. Users can easily access a thorough, systematic analysis of sequence–structure relationships (Figure 1) based on a curated database of 201 protein dimers obtained from the Membrane Proteins of Known 3D structure (MPSTRUC) (18). MENSAdb delivers tabular and graphical data formats that can be visually explored for a large number of MP features based on conservation, accessible solvent area (ASA) descriptors, average *B*-factors, intermolecular contacts at 2.5 Å and 4.0 Å distance cut-offs, hydrophobic contacts, hydrogen bonds, salt bridges, π–π stacking, T-stacking and cation–π interactions. Additionally, users can inspect differences in these features between three distinctive residue classes: (i) non-surface, (ii) surface and non-interfacial and (iii) surface and interfacial. The web server relies on a custom front-end application that provides the results to the user. The resulting knowledge and full database can be easily assessed and downloaded.

Our main goal with the integration of these features into a single platform is to assist the development of prediction models associated with MPs, either for classification or for regression tasks, as well as to help researchers to better understand MP interfacial characteristics. Our database is freely available at www.moreiralab.com/resources/mensadb.

### Materials and methods

#### Data collection and pre-processing

Experimental structures of 167 unique transmembrane (TM) proteins that included β-barrel TMs and α-helix TMs were obtained from MPSTRUC (http://blanco.biomol.uci.edu/mpstruc/) (18). These correspond to structures achieved mainly from X-ray crystallography (91%) or electron microscopy (4%), with a resolution below or equal to 4.50 Å, and less frequently from nuclear magnetic resonance (5%). We discarded all non-TM, monomeric and monotopic (not embedded in the lipid bilayer) proteins. Pre-processing of the database was performed by excluding dimers in which one of the chains was a soluble protein, single MPs interacting with small soluble peptides (protein–peptide), pores, protein–antibodies (since antibodies are soluble proteins) and proteins with small organic or non-organic ligands (protein–ligand). In the previous case, the complex was maintained if the presence of more than one MPs chain was observed. Additionally, structures with unknown residues or with many incomplete amino acids were also excluded, as were structures with interfaces interacting highly with lipids. Sequences were filtered to ensure at most 35% sequence redundancy in each interface by using the PISCES web server (19). The final database was composed of 63% (*n* = 105/167) homodimers and 37% (*n* = 62/167) heterodimers. From the Protein Data Bank (PDB) files, all possible dimer combinations were extracted for the structures in which the number of chains was higher than two (functional high-order oligomers) and it is constituted by 201 protein dimer combinations (Supplementary File 1). The selected structures were then subjected to further processing. In particular, we (i) identified and removed residues outside the TM domain according to the MPSTRUC (18) annotation of α-helix and β-barrel amino acids available in the PDB (20) in conjunction with visual inspection; (ii) removed unnecessary heteroatoms; (iii) reversed mutated non-standard amino acids (e.g. selenomethionine was mutated to methionine); and (iv) added hydrogens to the structures. In-house PyMOL (20) and Visual Molecular Dynamics (VMD) scripts (21) were used to perform these pre-processing steps.

#### Definition of interfacial and non-interfacial residues

The relative solvent accessibility (RSA) defined as the ratio between an amino acid ASA value and its corresponding area in a Gly-X-Gly peptide was calculated using an in-house pipeline with Database of Secondary Structure assignments for all Proteins entries (DSSP) (22). Residues above a 0.20 RSA cut-off were considered as surface residues (23). We obtained 55 008 possible surface residues from a total of 91 861, while the remaining ones were considered core residues. Secondly, we considered those for which the pairwise distance between any atom of chain A and any atom of chain B was below 5 Å as interfacial residues, splitting surface residues into two classes: interfacial (15 277 residues) and non-interfacial ones (39 731 residues).

#### Determination of sequence and structural features of all residues

Evolutionary conservation of all sites was calculated using the Jensen–Shannon divergence (JSD) measure, a symmetrized and smoothed version of the Kullback–Leibler divergence (24), of the Position-Specific Scoring Matrix (PSSM), which itself was calculated with a local deployment of PSI-BLAST against the NCBI non-redundant database with parameters num_iterations = 3 and evalue = 0.001 (25). Equation 1 was used to quantify the similarity between two probability distributions and compares the amino acid distribution observed in PSSM ${p_{ia}}$ with a background distribution ${f_a}$.

(1) \begin{equation*}JSD = H\left( {{{{p_{ia}} + {p_a}} \over 2}} \right) - {1 \over 2}H({p_{ia}}) - {1 \over 2}H({f_a})\end{equation*}

*H*(^.^) denotes the entropy of amino acid distribution. The code provided by Capra *et al.* was introduced into the pipeline due to its high performance in comparison with other methods (16). This metric works on the premise that the highest JSD value corresponds to a more conserved residue. We tested three different background distributions, BLOSUM62 (the PSI-BLAST default one), SLIM (26) and bbTM (27) to assess which one of them was the most suitable for MPs interface prediction. SLIM is a non-symmetric matrix optimized for TM protein segments, whereas bbTM is a set of matrices optimized for β-barrel proteins that uses three different matrices (one for intracellular segments, one for extracellular segments and another for TM residues). Herein, we only used the matrix developed for TM segments, since the remaining residues were already excluded from the analysis. We also generated a new column named ‘appropriate JSD’ in which we selected SLIM and bbTM depending on the presence and absence of an α-helix or β-barrel protein, respectively.

DSSP was used to calculate the RSA not only in the complexed form but also in the monomeric form, which were then multiplied by Sander and Rost amino acid constants (ALA: 106, ARG: 248, ASN: 157, ASP: 163, CYS:135, GLN: 198, GLU: 194, GLY: 84, HIS: 184, ILE: 169, LEU: 165, LYS: 205, MET: 188, PHE: 197, PRO: 136, SER: 130, THR: 142, TRP: 227, TYR: 222 and VAL: 142) (28) to calculate ASA of each amino acid, ‘i’, in the complexed (_comp_ASA_i_) and monomeric (_mon_ASA_i_) systems, respectively. These values were also used to calculate ΔASA_i_ (Equation 2).

(2) \begin{equation*}\Delta AS{A_i} = comp AS{A_i} - mon\,\,AS{A_i}\end{equation*}

For further clarification, we also listed all _rel_ASA_i_ values (Equation 3), which allows the differentiation of residues with equal ΔASA_i_ but with different absolute monomer ASA values (29–31).

(3) \begin{equation*}relASA_i = \frac{\Delta ASA_i}{monASA_i}\end{equation*}

We also extracted the temperature factor (*B*-factor) value for each residue from the PDB file of the analysed structures (obtained directly from MPSTRUC) using Biopython (32).

#### Determination of structural descriptors of MP–protein interface

Close and hydrophobic contacts, hydrogen bonds, salt bridges and π-interactions (π–π stacking, T-stacking and cation–π interactions) were described using BINANA—Binding Analyzer, a Python-implemented algorithm that characterizes protein complexes (33). Close contacts correspond to the number of pairs of atoms formed within 2.5 and 4.0 Å radius.

#### Data treatment

Since the composition of the database was not equally distributed across the three classes of MPs presented here, we defined a correction factor (C*_factor_*), Equation 4, based on the concept of propensity score calculation, as shown by Huang (34). This factor is defined as the ratio between the frequency of occurrence of residue *i* in each one of the classes (*fi*_CLAS_) and the frequency of occurrence of the total number of amino acids in that class (*fi*_TOT_). The obtained MP-class-specific C*_factor_* was used to correct the various metrics described in the ‘Results’ section by multiplying them by their respective C*_factor_* except that of _rel_ASA.

(4) \begin{equation*}{C_{factor}} = {{f{i_{CLAS}}} \over {f{i_{TOT}}}}\end{equation*}

#### Statistics

For all plots, residues are ordered by increasing hydrophobicity based on the Kyte and Doolittle hydropathy index (35). Descriptive statistics such as three quartiles (Q1, Q2 and Q3), average and standard deviation were obtained using Pandas, a Python library (36). *P*-values were calculated through SciPy (https://docs.scipy.org/) with the independent *t*-test and one-way ANOVA. Further statistics were calculated for amino acids sets split according to the hydrophilic and hydrophobic potential as (i) charged—Asp, Glu, Lys and Arg; (ii) positively charged—Lys and Arg; (iii) negatively charged—Asp and Glu; (iv) polar—Ser, Thr, Asn, Gln, Tyr and His; (v) non-polar—Ala, Val, Ile, Leu, Met, Phe and Trp; aromatic—Phe, Trp and Tyr. Cys, Gly and Pro were not included in those subsets.

#### Code availability

MENSAdb code used for all the structural and physicochemical analyses of MP dimers is freely distributed as a GitHub repository at https://github.com/MoreiraLAB/mensadb-open. The available Python code allows users to perform feature extraction using a pre-processed PDB file easily. For detailed information on all the pre-processing steps (trimming of non-TM residues, removal of heteroatoms, mutation of exotic residues, modelling of incomplete structures and dimer extraction from the structure files), please see Preto *et al.* (37). The addition of hydrogens was implemented within the pipeline available in the GitHub repository. The original code was tested in a 64-bit version of Linux Ubuntu 18.04 (Intel Xeon 40 Core 2.2 GHz, 126 GB RAM) and required the installation of Python version 3.7.2 with the following free and open-source packages: NumPy ≥ 1.16.1, pandas ≥ 0.23.4, vmd-python ≥ 3.0.6, dit ≥ 1.2.3, Biopython ≥ 1.7.3 and standalone software: BLAST+ ≥ 2.9.0, BINANA ≥ 1.2.0, DSSP ≥ 3.0.7, MGTools ≥ 1.5.6 and AutoDock ≥ 3.0.7. The JSD measure we determined using a non-redundant protein database for comparison (for download options, please see https://ftp.ncbi.nlm.nih.gov/blast/db/).

#### Database development

Data resulting from this work are available through MENSAdb (www.moreiralab.com/resources/mensadb), without the need for login, registration or license, a rich data visualization web application built using Python’s ‘Flask’-based ‘Dash’ visualization framework (by ‘Plotly’). MENSAdb’s real-time query features are supported by a MongoDB back end, which enables the application to query, filter and aggregate the data in multiple meaningful ways. To boost performance, a ‘Flask’ caching layer is applied to support the complex queries required for visualization. To further ensure performance and security and support high-availability scenarios, all HTTP traffic directed at MENSAdb is served by the NGINX high-performance webserver and load balancer, which then routes it to multiple MENSAdb application instances. The final database of MENSAdb containing all the raw data of structural and physicochemical properties of MPs is publicly available from Figshare (Data Citation 1; dx.doi.org/10.6084/m9.figshare.7808909), and the full membrane dimer structures listed according to PDB code can be found in Supplementary File 1.

## Results and discussion

### MP dimer composition and characteristics

**Figure 2.**
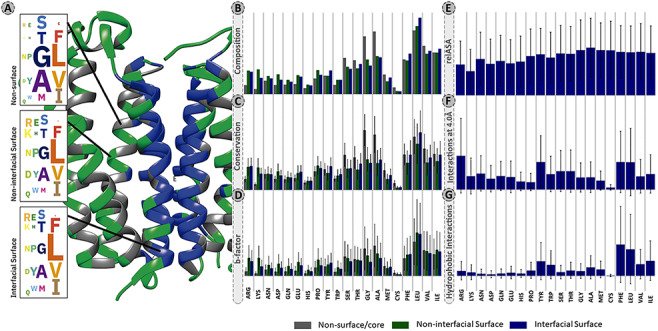
Panel of selected structural and physicochemical properties of MPs and their interactions. (A)—residue distribution of the translocator membrane protein (PDBid: 4UC1) from *Rhodobacter sphaeroides* (61). Amino acids are coloured according to the protein region within which they are embedded: grey—non-surface residues; green—non-interfacial surface residues; blue—interfacial surface residues. (B)—residue composition of the database. The correction factor described in section “Data treatment” of Material and methods was not applied here. (C)—normalized evolutionary conservation scores. (D)—normalized B-factor scores. (E)—normalized _rel_ASA. (F)—normalized intermolecular contacts at 4 Å. (G)—normalized hydrophobic contacts.

The overall residue distribution in Figure 2A and B shows that MPs have a higher content of hydrophobic and aromatic residues, such as leucine (13.2%), alanine (9.4%), valine (8.6%), glycine (8.4%), isoleucine (8.3%) and phenylalanine (6.9%) that account for 54.8% of all detected residues. For a better clarification the percentages presented in this sub-section, oppositely to remaining subsections are listed without correction factor. Indeed, these residues were shown to contribute the most to the accuracy of machine learning (ML) models developed for predicting protein–protein binding sites (38). This high content in hydrophobic residues, also previously reported in other studies (14, 38–43), is essential since it favours the thermodynamic interactions with the lipid bilayer. Figure 2A and B also show that GAS residues are significantly enriched at the MPs core (12.3%) and non-interfacial surface locations (8.5%), in comparison to interfacial surface (3.0%). These small residues are the strong driving force for membrane folding (44, 45). As expected, charged residues (arginine, aspartate, glutamate and lysine) are typically excluded from the MPs interface (surface: 7.4%; core: 2.6%; interface: 2.3%).

**Figure 3.**
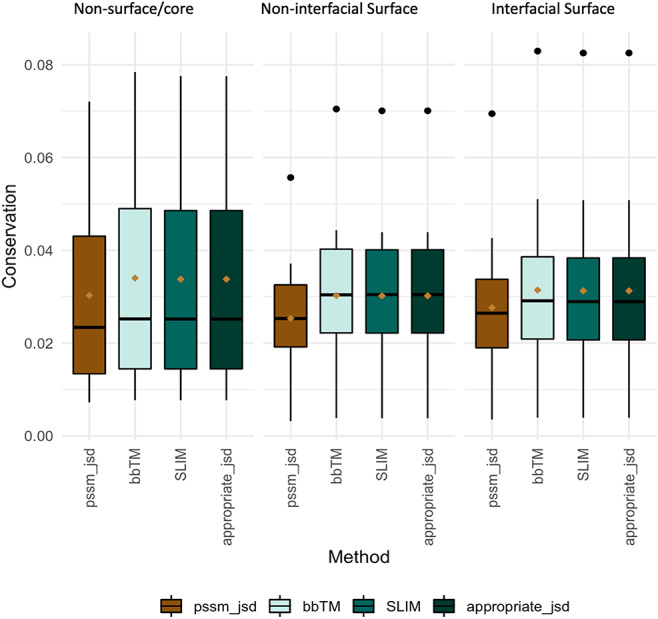
Conservation JSD distribution using BLOSUM62, SLIM, bbTM and the appropriate JSD background matrices (SLIM and bbTM were considered for α-helix and β-barrel proteins, respectively). Mean values are represented as a brown diamond. The results from the multiple pairwise test against all three background matrices yielded non-significant.

Evolutionary conservation of protein sequences is a key feature to better understand and characterize the functionally and structurally important residues at PPIs. Herein, we used three different background matrices to calculate conservation, namely BLOSUM62 (PSSM_JSD), SLIM and bbTM as well as the ‘appropriate_JSD’. Figure 3 illustrates their distribution split into three different protein regions: core/non-surface, interfacial surface and non-interfacial surface. The three different background matrices yielded similar results, which were non-significant according to multiple pairwise test. The same pattern was observed for all, with conservation being lower for surface, followed by interface and then protein core. As the used background matrix does not change the main conclusions about conservation at a MP dimer, we decided to follow up with the BLOSUM62 matrix for an easier implementation by the reader. Figure 2C reveals that for MPs, the more conserved JSD normalized values were found in the non-surface (0.05 ± 0.03) and in the interface (interface: 0.04 ± 0.02, surface non-interfacial 0.03 ± 0.02). The highest differences were for the GAS residues of the core region (core: 0.06 ± 0.03, surface: 0.03 ± 0.02; interface: 0.03 ± 0.02) and for the non-polar residues at protein core (core: 0.05 ± 0.02; surface: 0.04 ± 0.02; interface: 0.05 ± 0.03). These results, albeit not remarked different, support that the core and the interface are the most conserved regions, granting the necessary structural stability at specific PPIs, as previously observed (46). Additional results are available in the ‘Conservation’ option in the MENSAdb webserver.

*B*-factor (Figure 2D), related to the displacement of an atom from its reference position due to thermal motion and positional disorder (47), is typically used in a variety of applications including as a measure of atoms mobility for PPIs prediction (48, 49). We observed a decrease in normalized average *B*-factor values of the interfacial residues compared to the non-interfacial surface ones (5.71 ± 6.10 Å^2^ vs 6.25 ± 6.16 Å^2^), putting their average closer to the non-surface MP residues (6.02 ± 5.69 Å^2^). Also, positively charged residues are one of the most dissimilar ones (*B*-factor core: 1.19 ± 0.96 Å^2^; *B*-factor surface: 5.34 ± 3.72 Å^2^; *B*-factor interface: 3.74 ± 2.86 Å^2^). This is in agreement with the fact that residues participating in PPIs are usually less flexible in comparison with the ones from the surface, which is reflected in lower *B*-factor values (49–51). Leucine, very conserved at the interface, seems to also have a higher mobility at PPI-associated locations (surface: 12.66 ± 9.86 Å^2^; interface: 12.25 ± 9.52 Å^2^ vs core: 9.88 ± 6.83 Å^2^). Previous studies have suggested that leucine and isoleucine have an important role in flexible loop-mediated PPIs (52). Users can find illustrative plots of average *B*-factor values (by residue) in the ‘Average *B*-factor’ option in the MENSAdb web server.

The ASA descriptors detect protein regions that, when interacting or aggregating, lose solvent accessible area, while relASA indicates the relative exposed solvent surface area. MENSAdb and Figure 2E show that _rel_ASA, which is the fraction of ΔASA by _mon_ASA, is increased upon complex formation. These seem to be particularly relevant for non-polar residues (core: 5.27 ± 19.78 Å^2^; surface: 0.00 ± 0.09 Å^2^; interface: 52.01 ± 32.49 Å^2^). Additional and detailed information about ‘Monomer Accessible Surface Area’ (_mon_ASA), ‘Complex Accessible Surface Area’ (_comp_ASA), ‘Delta Accessible Surface Area’ (ΔASA) and ‘Relative Accessible Surface Area’ (_rel_ASA) can be viewed in MENSAdb web server options.

### Characteristics of interfacial residues

Identification and characterization of critical features of membrane PPI dimers can provide important clues to pinpoint residues or interactions, important for drug development. For this, additional interfacial structural characteristics were quantified to better understand MP dimers. Concerning the intermolecular atomic contacts per amino acid type, we observed that the aromatic residues (Figure 2F, corrected contacts at 4 Å: 0.56 ± 0.61) are much more prone to establish close contacts at short distance than other residues. Arg was also highlighted in our results (corrected contacts at 4 Å: 0.75 ± 0.82). For further information, check the ‘Interactions at 2.5 Angstroms’ and ‘Interactions at 4.0 Angstroms’ options in the MENSAdb web server.

Hydrophobicity involving large aromatic residues is key in MP dimers and aromatic residues, and non-polar residues show a high number of hydrophobic contacts (Figure 2G, aromatic: 0.25 ± 0.34 and non-polar: 0.23 ± 0.32). In particular, Phe and Tyr establish π–π stacking, T-stacking and cation–π interactions in different dimers. Cation–π interactions are also particularly relevant for Arg (for a closer detailed view, please see the ‘Hydrophobic Interactions’, ‘Pi–Pi Interactions’, ‘T-Stacking Interactions’ and ‘Cation–Pi Interactions’ options in the MENSAdb).

Additionally, although MP residues reside in a non-polar (low dielectric) environment (8, 53), both salt bridges between charged residues and hydrogen bonds through almost all amino acids are common to stabilize the interface and promote complex formation. Hydrogen bonds measured here involving both side chains and backbone are particularly important not only for charged residues (0.01 ± 0.03) but also for aromatic ones (0.01 ± 0.02), in particular tyrosine (0.01 ± 0.03) and tryptophan (0.01 ± 0.01). For a closer detailed view, please see the ‘Salt-bridge Interactions’ and ‘Hydrogen-bond Interactions’ options in the MENSAdb web server.

All different values presented herein showed statistical relevance.

All the results presented herein were obtained under the assumption that the interfaces in this study were biologically relevant, and utmost care was taken to ensure this (Supplementary File 1). Further limitations could arise from possible crystallographic artefacts.

### MENSAdb interface and usability

**Figure 4.**
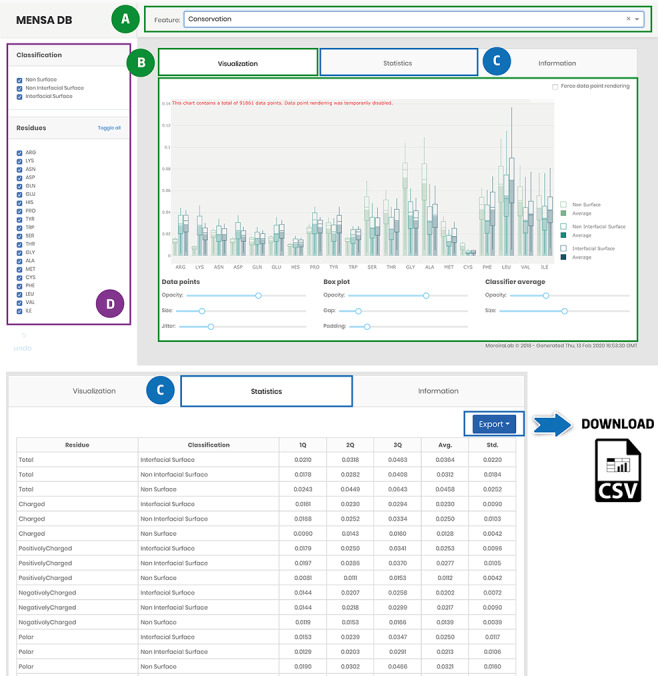
Main landing page of MENSAdb web server. Screenshot of the home page (A)—quickly query by evolutionary or physicochemical features. (B)—In the visualization tab, the results are shown in a graphical format. Users can easily change visual properties (opacity, size, jitter, gap and padding) by interacting with the lower panel. (C)—Statistics tab displays the data in a tabular format with associated metrics (Q1, Q2, Q3, Average-Avg. and Standard Deviation-Std.). Stats and raw data can be downloaded using the Export button in the top right corner, as a .csv file. (D)—In the left panel, users can filter graphic data by classification and residue type.

The developed application enables users to explore the MP-dimer database (Figure 4). Access to evolutionary and physicochemical features is provided through a drop-down menu on the main page (Figure 4A). The data are presented in downloadable box plots for visual inspection that can be easily changed, for example, by filtering, zooming or panning (Figure 4B). Besides, data-associated statistics are also accessible in a tabular format [Q1, Q2, Q3, Average (Avg.) and Standard Deviation (Std.)] (Figure 4C). Stats and raw data can be downloaded as a .csv file using the export button for further reuse and integration in other studies. Users can also filter data for each selected feature by classification (non-surface, non-interfacial surface and interfacial surface) or residue type (Figure 4D). The database also has an ‘Information’ tab with general information for each included feature and a brief description of the underlying methods for their acquisition and pre-processing to help first-time users. MENSAdb will continue to be updated at least annually, and we expect, shortly, to integrate a new model for the prediction of MP interfaces.

MENSAdb is the first comprehensive resource dedicated explicitly to exposing the evolutionary and physicochemical features of dimeric MP structures. Our main goal with the integration of these features into a single platform is to assist the development of experimental and computational assays, relevant for a better understanding of dimeric MP interactions and interfaces of this largest but poorly studied type of proteins. In the last years, some studies used evolutionary and physicochemical properties similar to the ones provided in our database to train ML for the prediction of MP complex binding sites (38, 46, 54). Nevertheless, as far as we know, herein we offer original features such as the ones from membrane PPI analysis not yet used or provided by other databases more dedicated to MP structures (PDBTM, OPM, MemProtMD and, MPSTRUC) or classification (TCDB).

## Supplementary Material

baab013_Supp

## Data citation

Matos-Filipe,P., Preto,A.J., Koukos,P.I., Mourão,J., Bonvin,A.M.J.J., Moreira,I.S. *figshare* dx.doi.org/10.6084/m9.figshare.7808909.
