# Supplementary material for: MENSAdb: a thorough structural analysis of membrane protein dimers
Source: Database (Oxford). 2021 Apr 5;2021:baab013. doi: 10.1093/database/baab013 (PMC8023553; doi:10.1093/database/baab013)
Supplement: baab013_Supp [file baab013_supp.zip › suppl_data/MENSAdb_SI_R1-20-01-2021.docx]

***Supplementary Information***

**Supplementary File 1**

**Table 1.** Full membrane dimer dataset used in MENSAdb, discriminated by PDB code.

| ***PDB CODE*** | **DIMER CHAINS** | **CLASS** | **NAME** | **SPECIES** | **TAXONOMIC DOMAIN** | **RESOLUTION** | **UNIQUE**  **CHAINS** | | | **SUBGROUP NAME** | **COMPLEX CLASS** | **OLIGOMER STATE** | **STOICHIOMETRY** | **Excluded residues** |
| --- | --- | --- | --- | --- | --- | --- | --- | --- | --- | --- | --- | --- | --- | --- |
| *1A0T* | PQ | beta | ScrY sucrose-specific porin | *Salmonella* Typhimurium | Bacteria | 2.40 | 1 | | | Beta-Barrel Membrane Proteins: Porins and Relatives | protein-ligand | multimer | ['BA1: Homo 3-mer - A3', 'Asymmetry: C3'] |  |
| *1BL8* | AB | alpha | KcsA Potassium channel, H^+^ gated | *Streptomyces lividans* | Bacteria | 3.20 | 1 | | | Channels: Potassium, Sodium, & Proton Ion-Selective | protein-protein | multimer | ['Asymmetry: C4', 'BA1: Homo 4-mer - A4'] |  |
| *1EK9* | AB | beta | TolC outer membrane protein | *Escherichia coli* | Bacteria | 2.10 | 1 | | | Beta-Barrel Membrane Proteins: Monomeric/Dimeric | protein-protein | multimer | ['BA1: Homo 3-mer - A3', 'Asymmetry: C3'] | A:1-3,5-39,79-245,296-296,298-357,359-428; B:1-3,5-39,77-77,79-245,296-296,298-357,359-428 |
| *1EYS* | HM, LM | alpha | Photosynthetic Reaction Center | *Thermochromatium tepidum* | Bacteria | 2.20 | 4 | | | Photosynthetic Reaction Centers | protein-protein | multimer | ['Asymmetry: C1', 'BA1: Hetero 4-mer - ABCD'] | M:305-318; H:40-43,59-259 |
| *1EZV* | CD, CG | alpha | Cytochrome bc | *Saccharomyces cegy5brevisiae* | Eukaryota | 2.30 | 13 | | | Electron Transport Chain Complexes: Complex III | protein-antibody | multimer* | ['Asymmetry: C1', 'BA1: Hetero 20-mer - A2B2C2D2E2F2G2H2I2JK'] | A:386-456; B:17-368; D:62-257,300-306 |
| *1KF6* | CD | alpha | E. coli Quinol-Fumarate Reductase with Bound Inhibitor HQNO | *Escherichia coli* (strain K12) | Bacteria | 2.70 | 8 | | | Oxidoreductases | protein-protein | multimer | ['Asymmetry: C1', 'BA1: Hetero 4-mer - ABCD', 'BA2: Hetero 4-mer - ABCD', 'BA3: Hetero 8-mer - A2B2C2D2'] | C:1-8; D:0-3 |
| *1L7V* | AB | alpha | BtuCD Vitamin B Transporter | *Escherichia coli* | Bacteria | 3.20 | 2 | | | ATP Binding Cassette (ABC) Transporters | protein-protein | multimer* | ['Asymmetry: A2B2', 'BA1: Hetero 4-mer - A2B2'] |  |
| *1LNQ* | AB | alpha | MthK Potassium channel, Ca ^++^ gated | *Methanothermobacter thermautotrophicus* | Archaea | 3.30 | 1 | | | Channels: Potassium, Sodium, & Proton Ion-Selective | protein-protein | multimer | ['Asymmetry: A8', 'BA1: Homo 8-mer - A8'] | A:116-336; B:116-336 |
| *1MAL* | AB | beta | LamB Maltoporin | *Escherichia coli* | Bacteria | 3.10 | 1 | | | Beta-Barrel Membrane Proteins: Porins and Relatives | protein-protein | multimer | ['BA1: Homo 3-mer - A3', 'Asymmetry: A3'] |  |
| *1NEK* | CD | alpha | Succinate: quinone oxidoreductase (SQR, Complex II) | *Escherichia coli* | Bacteria | 2.60 | 4 | | | Electron Transport Chain Complexes: Complex II | protein-protein | multimer* | ['Asymmetry: BA1', 'BA1: Hetero 4-mer - ABCD', ‘BA2: Hetero 12-mer - A3B3C3D3’, ‘BA3: Hetero 8-mer - A2B2C2D2’] | C:1-16 |
| *1OCC* | AB, AC, AE, AL, AM, BI, CG, CJ, DK, LM | alpha | Cytochrome C Oxidase | *Bos taurus* (bovine) heart mitochndria | Eukaryota | 2.80 | 13 | | | Electron Transport Chain Complexes: Complex IV (Cytochrome C Oxidase) | protein-protein | multimer | ['Asymmetry: A2B2C2D2E2F2G2H2I2J2K2L2M2', 'BA1: Hetero 26-mer - A2B2C2D2E2F2G2H2I2J2K2L2M2'] | B:92-227; I:1-7; D:4-72 |
| *1OTS* | AB | alpha | H^+^ /Cl^-^ Exchange Transporter | *Escherichia coli* | Bacteria | 2.51 | 5 | | | Hcl Exchange Transporters | protein-antibody | multimer* | ['Asymmetry: A2B2C2', 'BA1: Hetero 6-mer - A2B2C2'] |  |
| *1P7B* | AB | alpha | KirBac1.1 Inward-Rectifier Potassium channel (closed state) | *Burkholderia pseudomallei* | Bacteria | 3.65 | 1 | | | Channels: Potassium, Sodium, & Proton Ion-Selective | protein-protein | m-m | ['Asymmetry: Homo 4-mer - A4'] | A:153-195,206-289,296-309; B:153-195,206-289,296-309 |
| *1PRC* | LM | alpha | Photosynthetic Reaction Center | *Blastochloris viridis* | Bacteria | 2.30 | 4 | | | Photosynthetic Reaction Centers | protein-protein | multimer | ['Asymmetry: ABCD', 'BA1: Hetero 4-mer - ABCD', 'BA2: A2B2C2D2'] | M:306-323 |
| *1RHZ* | AB, AC | alpha | SecYE β - translocon | *Methanococcus jannaschii* | Archaea | 3.50 | 3 | | | Sec and Translocase Proteins | protein-peptide | both | ['Asymmetry: ABC', 'BA1: Hetero 3-mer - ABC'] |  |
| *1UUN* | AB | beta | MspA mycobacterial porin | *Mycobacterium smegmatis* | Bacteria | 2.50 | 1 | | | Beta-Barrel Membrane Proteins: Porins and Relatives | protein-protein | m-m | ['BA1: Homo 8-mer - A8', 'Asymmetry: A2'] | A:1-70,124-184; B:1-69,125-184 |
| *1XFH* | AB | alpha | Glutamate Transporter Homologue (Glt) | *Pyrococcus horikoshii* | Archaea | 3.50 | 1 | | | Amino Acid Secondary Transporters | protein-protein | multimer | ['Asymmetry: A3', 'BA1: Homo 3-mer - A3'] |  |
| *1YEW* | AB, BC | alpha | Particulate methane monooxgenase (pMMO) | *Methylococcus capsulatus* | Bacteria | 2.80 | 3 | | | Oxygenases | protein-protein | multimer | ['Asymmetry: C1', 'BA1: Hetero 3-mer - ABC', 'BA2: Hetero 3-mer - ABC', 'BA3: Hetero 3-mer - ABC'] | A:33-179,267-414; C:204-230 |
| *1ZLL* | AB | alpha | Phospholamban homopentamer | *Homo sapiens* | Eukaryota | NMR Structure |  | | 2 | P-type ATPase | protein-protein | multimer | ['Asymmetry: C5', 'BA1: Homo 5-mer - A5’] | A:1-16; B:1-15 |
| *1ZOY* | CD | alpha | Succinate:ubiquinone oxidoreductase (SQR, Complex II; pig heart) | *Sus scrofa* | Eukaryota | 2.40 | 4 | | | Electron Transport Chain Complexes: Complex II | protein-protein | multimer* | ['Asymmetry: ABCD', 'BA1: Hetero 4-mer - ABCD'] | A:10-29,139-622; B:9-138,241-247 |
| *1ZRT* | CD, CP | alpha | Cytochrome bc | *Rhodobacter capsulatus* | Bacteria | 3.50 | 3 | | | Electron Transport Chain Complexes: Complex III | protein-protein | multimer | ['Asymmetry: A2B2C2', 'BA1: Hetero 6-mer - A2B2C2'] | D:2-161,164-168,172-216 |
| *2AHY* | AB | alpha | NaK channel (Na^+^ complex) | *Bacillus cereus* | Bacteria | 2.40 | 1 | | | Channels: Potassium, Sodium, & Proton Ion-Selective | protein-protein | m-m | ['Asymmetry: Homo 4-mer - A4'] |  |
| *2BL2* | AB | alpha | Rotor of V-type Na^+^ -ATPase | *Enterococcus hirae* | Bacteria | 2.10 | 1 | | | Bacterial V-type ATPase | protein-protein | multimer | ['Asymmetry: Homo 10-mer - A10'] |  |
| *2BS2* | CF | alpha | Fumarate Reductase Complex | *Wolinella succinogenes* | Bacteria | 1.78 | 3 | | | Electron Transport Chain Complexes: Complex II | protein-protein | multimer | ['Asymmetry: A2B2C2', 'BA1: Hetero 6-mer - A2B2C2'] |  |
| *2FBW* | CD | alpha | Succinate: ubiquinone oxidoreductase (SQR, Complex II; chicken heart) w. carboxin inhibitor | *Gallus gallus* | Eukaryota | 2.10 | 4 | | | Electron Transport Chain Complexes: Complex II | protein-protein | multimer* | ['Asymmetry: C1’, ‘BA1: Hetero 4-mer – ABCD’, ‘BA2: Hetero 4-mer – ABCD’] | C:2-29 |
| *2FYN* | AB, AD | alpha | Cytochrome bc | *Rhodobacter sphaeroides* | Bacteria | 3.20 | 3 | | | Electron Transport Chain Complexes: Complex III | protein-protein | multimer | ['Asymmetry: A6B6C6', 'BA1: Hetero 6-mer - A2B2C2', 'BA2: A2B2C2', 'BA3: A2B2C2'] | A:429-430; B:1-256 |
| *2GR8* | AC | beta | Trimeric autotransporter | *Haemophilus influenzae* | Bacteria | 2.00 | 1 | | | Outer Membrane Autotransporters | protein-protein | multimer | ['BA1: Homo 3-mer - A3', '2': 'A3', 'Asymmetry: A6'] |  |
| *2HAC* | AB | alpha | Transmembrane dimer of the TCR-CD3 complex | *Homo sapiens* | Eukaryota | NMR Structure | | 3 | | Immune Receptors | protein-protein | m-m | ['Asymmetry: C2', 'BA1: Homo 2-mer – A2’] | A:(-3) - (-1),4; B:(-3) - (-1),4 |
| *2HYD* | AB | alpha | Sav1866 Multidrug Transporter | *Staphylococcus aureus* | Bacteria | 3.00 | 1 | | | ATP Binding Cassette (ABC) Transporters | protein-ligand | m-m | ['Asymmetry: A2', 'BA1: Homo 2-mer - A2'] | A:323-578; B:324-578 |
| *2J1N* | AB | beta | OmpC Osmoporin | *Escherichia coli* | Bacteria | 2.00 | 1 | | | Beta-Barrel Membrane Proteins: Porins and Relatives | protein-protein | multimer | ['BA1: Homo 3-mer - A3', 'Asymmetry: A3'] |  |
| *2J8S* | AB | alpha | Drug Export Pathway of Multidrug Exporter AcrB Revealed by DARPin Inhibitors | *Escherichia coli* | Bacteria | 2.54 | 3 | | | Membrane protein: exporter | protein-protein | m-m | ['Asymmetry: Hetero 5-mer - A3B2','BA1: Hetero 5-mer - A3B2'] | A:34-324,563-869; B:34-326,563-865 |
| *2K9Y* | AB | alpha | EphA2 transmembrane segment dimer | *Homo sapiens* | Eukaryota | NMR Structure | 1 | | | Erythropoietin-Producing Hepatocellular Receptors | protein-protein | m-m | ['Asymmetry: Homo 2-mer - A2'] | A:523-533,559-563; B:523-533,559-563 |
| *2KIX* | AB | alpha | M2 proton channel (BM2) | Influenza B | Viruses | NMR Structure | 1 | | | Channels: Other Ion Channels | protein-protein | multimer | ['Asymmetry: C4', 'BA1: Homo 4-mer – A4’] |  |
| *2L35* | AB | alpha | DAP12 dimeric signaling domain in complex with activating receptor NKG2C | *Homo sapiens* | Eukaryota | NMR Structure | 2 | | | Immune Receptors | protein-protein | multimer | ['Asymmetry: C1', 'BA1: Hetero 2-mer – AB’] |  |
| *2LZL* | AB | alpha | FGFR3 Fibroblast growth factor receptor 3 transmembrane dimer | *Homo sapiens* | Eukaryota | NMR Structure | 1 | | | Fibroblast Growth Factor Receptors | protein-protein | m-m | ['Asymmetry: Homo 2-mer - A2'] | A:357-371; B:357-371 |
| *2M59* | AB | alpha | VEGFR2 vascular endothelial growth factor receptor 2 transmembrane dimer | *Homo sapiens* | Eukaryota | NMR Structure | 1 | | | Vascular Endothelial Growth Factor Receptors | protein-protein | m-m | ['Asymmetry: Homo 2-mer - A2'] |  |
| *2MPN* | AB | alpha | YgaP rhodanese homodimeric transmembrane domain | *Escherichia coli* | Bacteria | NMR Structure | 1 | | | Rhodaneses | protein-protein | m-m | ['Asymmetry: Homo 2-mer - A2'] |  |
| *2MPR* | AB | beta | LamB Maltoporin | *Salmonella* Typhimurium | Bacteria | 2.40 | 1 | | | Beta-Barrel Membrane Proteins: Porins and Relatives | protein-protein | multimer | ['BA1: Homo 3-mer - A3', 'Asymmetry: A3'] | A:259-264; B:259-264 |
| *2NQ2* | AB | alpha | HI1470/1 Putative Metal-Chelate-type ABC Transporter | *Haemophilus influenzae* | Bacteria | 2.40 | 2 | | | ATP Binding Cassette (ABC) Transporters | protein-protein | multimer* | ['Asymmetry: A2B2', 'BA1: Hetero 4-mer - A2B2'] | A:38-50,142-145;B:35-53,140-146 |
| *2NWL* | AB | alpha | Aspartate Transporter Li^+^ -Bound State(Glt) | *Pyrococcus horikoshii* | Archaea | 2.96 | 1 | | | Amino Acid Secondary Transporters | protein-ligand | multimer | ['Asymmetry: A3', 'BA1: Homo 3-mer - A3'] | A:123-127; B:119-127 |
| *2O4V* | AB | beta | OprP phosphate-specific transporter | *Pseudomonas aeruginosa* | Bacteria | 1.90 | 1 | | | Beta-Barrel Membrane Proteins: Porins and Relatives | protein-ligand | multimer | ['BA1: Homo 3-mer - A3', 'Asymmetry: A3'] |  |
| *2ONK* | CD | alpha | Molybdate Transporter ModB Complexed with ModA | *Archaeoglobus fulgidus* | Archaea | 3.10 | 3 | | | ATP Binding Cassette (ABC) Transporters | protein-protein | multimer* | ['Asymmetry: A4B4C2', 'BA1: Hetero 5-mer - A2B2C', 'BA2: A2B2C'] |  |
| *2PNO* | AB | alpha | Leukotriene LTC Synthase in complex with glutathione | *Homo sapiens* | Eukaryota | 3.30 | 1 | | | Membrane-Associated Proteins in Eicosanoid and Glutathione Metabolism (MAPEG) | protein-ligand | multimer | ['Asymmetry: Homo 3-mer - A3', 'BA1: A3', 'BA2: A3', 'BA3: A3'] |  |
| *2Q7M* | AB | alpha | 5-Lipoxygenase-Activating Protein (FLAP) with Bound MK-591 Inhibitor | *Homo sapiens* | Eukaryota | 4.00 | 1 | | | Membrane-Associated Proteins in Eicosanoid and Glutathione Metabolism (MAPEG) | protein-ligand | multimer | ['Asymmetry: A6', 'BA1: Homo 3-mer - A3', 'BA2: A3'] |  |
| *2R6G* | FG | alpha | MalFGK -MBP Maltose uptake transporter complex | *Escherichia coli* | Bacteria | 2.80 | 4 | | | ATP Binding Cassette (ABC) Transporters | protein-protein | multimer* | ['Asymmetry: A2BCD', 'BA1: Hetero 5-mer - A2BCD'] | F:96-242,245-258  G:68-73 |
| *2VL0* | AB | alpha | Prokaryotic pentameric ligand-gated ion channel (ELIC) | *Erwinia chrysanthemi* | Bacteria | 3.30 | 1 | | | Cys-Loop Receptor Family | protein-protein | multimer | ['Asymmetry: A10', 'BA1: Homo 5-mer - A5', 'BA2: A5'] | A:11-195; B:11-194 |
| *2VPZ* | CG | alpha | Polysulfide Reductase PsrABC (native) | *Thermus thermophilus* | Bacteria | 2.40 | 3 | | | MGD Oxidoreductases | protein-protein | multimer* | ['Asymmetry: A2B2C2', 'BA1: Hetero 6-mer - A2B2C2'] | C:2-14; G:1-13 |
| *2WIE* | AB | alpha | Rotor of H^+^ -dependent F-ATP Synthase of an alkaliphilic cyanobacterium | *Spirulina platensis* | Bacteria | 2.10 | 2 | | | F-type ATPase | protein-protein | multimer | ['Asymmetry: Homo 15-mer - A15'] |  |
| *2WIT* | AB | alpha | BetP glycine betaine transporter | *Corynebacterium glutamicum* | Bacteria | 3.35 | 1 | | | Betaine/Choline/Carnitine Transporter (BCCT) Family | protein-ligand | multimer | ['Asymmetry: A3', 'BA1: Homo 3-mer - A3'] | A:273-274,558-589; B:273-274,559-589 |
| *2WLJ* | AB | alpha | KirBac3.1 Inward-Rectifier Potassium channel (semi-latched) | *Magnetospirillum magnetotacticum* | Bacteria | 2.60 | 1 | | | Channels: Potassium, Sodium, & Proton Ion-Selective | protein-protein | m-m* | ['Asymmetry: Homo 4-mer - A4'] | A:12-27,138-299; B:23-26,139-299 |
| *2X2V* | AB | alpha | Rotor of H^+^ -dependent F-ATP Synthase | *Bacillus pseudofirmus* OF4 | Bacteria | 2.50 | 1 | | | F-type ATPase | protein-protein | multimer | ['Asymmetry: A13', 'BA1: Homo 13-mer - A13'] | A:69; B:2-68 |
| *2XND* | OP | alpha | Fc-ring complex | *Bos taurus* | Eukaryota | 3.50 | 6 | | | F-type ATPase | protein-protein | multimer | ['Asymmetry: A8B3C3DEF', 'BA1: Hetero 17-mer - A8B3C3DEF'] |  |
| *2YEV* | AB, AC | alpha | Cytochrome C Oxidase, | *Thermus thermophilus* | Bacteria | 2.36 | 3 | | | Electron Transport Chain Complexes: Complex IV (Cytochrome C Oxidase) | protein-protein | multimer* | ['Asymmetry: C1', 'BA1: Hetero 3-mer – ABC’, 'BA2: Hetero 3-mer – ABC’] | B:115-337 |
| *2ZT9* | AB, AG, CH | alpha | Cytochrome complex | Nostoc sp. PCC 7120 | Bacteria | 3.00 | 8 | | | Electron Transport Chain Complexes: Cytochrome b | protein-protein | multimer | ['Asymmetry: Hetero 16-mer - A4B2C2D2E2F2G2'] | C:1-246 |
| *2ZW3* | AB | alpha | Connexin 26 (Cx26; GJB2) gap junction | *Homo sapiens* | Eukaryota | 3.50 | 1 | | | Channels: Gap Junctions | protein-protein | multimer | ['Asymmetry: A6', 'BA1: Homo 12-mer - A12'] | A:110-124; B:110-124 |
| *2ZXE* | AB, AG | alpha | Na,K-ATPase; shark | *Squalus acanthias* | Eukaryota | 2.40 | 3 | | | P-type ATPase | protein-protein | multimer* | ['Asymmetry: ABC', 'BA1: Hetero 3-mer - ABC'] | A:32-84,155-278,367-752; B:66-160,169-217,223-305 |
| *2ZY9* | AB | alpha | Improved crystal structure of magnesium transporter MgtE | *Escherichia coli* | Bacteria | 2.94 | 2 | | | membrane protein metal transport | protein-protein | m-m | ['Asymmetry: Homo 2-mer - A2','BA1: Homo 2-mer - A2',] | A:23-238; B:20-239 |
| *3A7K* | AB | alpha | Halorhodopsin (HR) | *Natronomonas pharaonis* | Archaea | 2.00 | 1 | | | Bacterial and Algal Rhodopsins | protein-protein | multimer | ['Asymmetry: A3', 'BA1: Homo 3-mer - A3'] |  |
| *3B07* | AB | beta | &gamma - hemolysin composed of LukF and Hlg2 | *Staphylococcus aureus* | Bacteria | 2.50 | 2 | | | Adventitious Membrane Proteins: Beta-sheet Pore-forming Toxins/Attack Complexes | protein-protein | multimer | ['Asymmetry: A4B4', 'BA1: Hetero 8-mer - A4B4'] | A:17-106,149-300; B:11-99,140-280 |
| *3B4R* | AB | alpha | Site-2 Protease (S2P). Intramembrane Metalloprotease | *Methanocaldococcus jannaschii* | Archaea | 3.30 | 1 | | | Intramembrane Proteases | protein-protein | m-m* | ['Asymmetry: A2', 'BA1: Homo 2-mer - A2'] |  |
| *3B60* | AB | alpha | MsbA Lipid flippase with bound AMPPNP | *Salmonella* Typhimurium | Bacteria | 3.70 | 1 | | | ATP Binding Cassette (ABC) Transporters | protein-protein | multimer | ['Asymmetry: Homo 2-mer - A2', 'BA1: A2'] | A:328-581; B:329-581 |
| *3D31* | CD | alpha | ModBC Molybdate ABC Transporter in a trans-inhibited state | *Methanosarcina acetivorans* | Archaea | 3.00 | 2 | | | ATP Binding Cassette (ABC) Transporters | protein-protein | multimer* | ['Asymmetry: A2B2', 'BA1: Hetero 4-mer - A2B2'] |  |
| *3DH4* | AB | alpha | vSGLT Sodium Galactose Transporter | *Vibrio parahaemolyticus* | Bacteria | 2.70 | 1 | | | Solute Sodium Symporter (SSS) Family | protein-ligand | multimer | ['Asymmetry: Homo 4-mer - A4', 'BA1: A2', 'BA2: A2'] | A:3-19; B:3-19 |
| *3DIN* | CD, CE | alpha | SecYEG translocon in complex with SecA | *Thermotoga maritima* | Bacteria | 4.50 | 4 | | | Sec and Translocase Proteins | protein-protein | multimer | ['Asymmetry: C1', ‘BA1: Hetero 4-mer – ABCD’, ‘BA2: Hetero 4-mer – ABCD’, ‘BA3: Hetero 4-mer – ABCD’] | C:42-61 |
| *3KLY* | AB | alpha | FocA formate transporter without formate | *Vibrio cholerae* | Bacteria | 2.10 | 1 | | | Channels : Formate/Nitrite Transporter (FNT) Family | protein-protein | multimer | ['Asymmetry: A5', 'BA1: Homo 5-mer - A5'] |  |
| *3MK7* | AB, AC | alpha | Cytochrome Oxidase | *Pseudomonas stutzeri* | Bacteria | 3.20 | 4 | | | Electron Transport Chain Complexes: Complex IV (Cytochrome C Oxidase) | protein-protein | multimer* | ['Asymmetry: C1', ‘BA1: Hetero 4-mer – ABCD’, ‘BA2: Hetero 4-mer – ABCD’] | B:41-202; C:87-303 |
| *3MP7* | AB | alpha | SecYE&beta translocon | *Pyrococcus furiosus* | Archaea | 3.10 | 2 | | | Sec and Translocase Proteins | protein-protein | m-m* | ['Asymmetry: AB', 'BA1: Hetero 2-mer - AB'] | A:91-113,139-144,202-204,261-280,277-280,371-399,401-406,446-470; B:7-26 |
| *3ND0* | AB | alpha | H^+^ /Cl^-^ Eukaryotic Exchange Transporter | *Synechocystis* sp. pcc 6803 | Bacteria | 3.20 | 1 | | | Hcl Exchange Transporters | protein-protein | m-m | ['Asymmetry: A2', 'BA1: Homo 2-mer - A2'] |  |
| *3O0R* | BC | alpha | Nitric Oxide Reductase | *Pseudomonas aeruginosa* | Bacteria | 2.70 | 6 | | | Nitric Oxide Reductases | protein-antibody | multimer* | ['Asymmetry: A2', 'BA1: Homo 2-mer - A2'] | C:48-146 |
| *3O44* | AB | beta | Cytolysin pore-forming toxin | *Vibrio cholerae* | Bacteria | 2.88 | 1 | | | Adventitious Membrane Proteins: Beta-sheet Pore-forming Toxins/Attack Complexes | protein-protein | multimer | ['Asymmetry: A14', 'BA1: Homo 7-mer - A7', 'BA2: A7'] | A:136-274,324-716; B:136-275,327-716 |
| *3ODU* | AB | alpha | CXCR4 chemokine receptor complexed with IT1t antagonist | *Homo sapiens* | Eukaryota | 2.50 | 1 | | | G Protein-Coupled Receptors (GPCRs) | protein-ligand | m-m* | ['Asymmetry: A2', 'BA1: Homo 2-mer - A2'] | A:900-901,1002-1161,1200-1201; B:900-901,1002-1161,1200-1201 |
| *3ORG* | AD | alpha | H^+^/Cl^-^ Eukaryotic Exchange Transporter | *Cyanidioschyzon merolae* | Eukaryota | 3.50 | 1 | | | Hcl Exchange Transporters | protein-protein | multimer | ['Asymmetry: A4', 'BA1: Homo 2-mer - A2', 'BA2: A2'] | A:535-600,656-710; D:533-600,656-710 |
| *3P5N* | AB | alpha | RibU, S Component of the Riboflavin Transporter | *Staphylococcus aureus* | Bacteria | 3.60 | 1 | | | Energy-Coupling Factor (ECF) Transporters | protein-ligand | m-m | ['Asymmetry: A2', 'BA1: Homo 2-mer - A2'] | A:142-152; B:142-152 |
| *3PJZ* | AB | alpha | TrkH potassium ion transporter | *Vibrio parahaemolyticus* | Bacteria | 3.51 | 1 | | | Superfamily of K Transporters (SKT proteins) | protein-protein | m-m | ['Asymmetry: A2', 'BA1: Homo 2-mer - A2'] | A:158-173; B:158-173 |
| *3QF4* | AB | alpha | Heterodimeric ABC exporter TM287-TM288 | *Thermotoga maritima* | Bacteria | 2.90 | 2 | | | ATP Binding Cassette (ABC) Transporters | protein-ligand | m-m* | ['Asymmetry: C1', 'BA1: Hetero 2-mer AB'] | A:-2-12,78-128,179-232,295-569; B:10-34,104-150,207-253,327-597 |
| *3QNQ* | AB | alpha | ChbC EIIC phosphorylation-coupled saccharide transporter | *Bacillus cereus* | Bacteria | 3.30 | 1 | | | Phosphoenolpyruvate-Dependent Phosphotransferases (PTSs) | protein-ligand | multimer | ['Asymmetry: A4', 'BA1: Homo 2-mer - A2', 'BA2: A2'] |  |
| *3RHW* | AB | alpha | GluCl&alpha; anion-selective receptor (Fab-invermectin complex) | *Caenorhabditis elegans* | Eukaryota | 3.26 | 5 | | | Cys-Loop Receptor Family | protein-antibody | multimer* | ['Asymmetry: A5B5C5', 'BA1: Hetero 15-mer - A5B5C5'] | A:132-340; B:1-131,263-340 |
| *3TDO* | AB | alpha | FNT3 Hydrosulphide Channel (HSC), pH 9.0 | *Clostridium difficile* | Bacteria | 2.20 | 1 | | | Channels : Formate/Nitrite Transporter (FNT) Family | protein-protein | multimer | ['Asymmetry: A5', 'BA1: Homo 5-mer - A5'] |  |
| *3TUI* | AB | alpha | Inward facing conformations of the MetNI methionine ABC transporter: CY5 native crystal form | *Escherichia coli* (strain K12) | Bacteria | 2.90 | 4 | | | HYDROLASE/TRANSPORT PROTEIN | protein-protein | m-m | ['Asymmetry: Hetero 8-mer - A4B4', ' BA1: Hetero 4-mer - A2B2', 'BA2: Hetero 4-mer - A2B2'] |  |
| *3UKM* | AB | alpha | Two-Pore Domain Potassium Channel (TWIK-1) | *Homo sapiens* | Eukaryota | 3.40 | 1 | | | Channels: Potassium, Sodium, & Proton Ion-Selective | protein-protein | multimer | ['Asymmetry: A4', 'BA1: Homo 2-mer - A2', 'BA2: A2'] | A:94-99,169-174; B:94-99,169-174 |
| *3UX4* | AB, AC | alpha | UreI proton-gated inner membrane urea channel | *Helicobacter pylori* | Bacteria | 3.26 | 1 | | | Channels: Urea Transporters | protein-ligand | multimer | ['Asymmetry: Homo 6-mer - A6'] | A:59-73; B:59-73; C:59-73 |
| *3VOU* | AB | alpha | NaK channel chimera with grafted C-terminal region of a NaV channel | *Bacillus weihenstephanensis* (NaK) and *Sulfitobacter pontiacus* (NaV) | Bacteria | 3.20 | 1 | | | Channels: Potassium, Sodium, & Proton Ion-Selective | protein-protein | m-m | ['Asymmetry: Homo 4-mer - A4'] | A:100-139; B:104-139 |
| *3VR8* | CD | alpha | Mitochondrial rhodoquinol-fumarate reductase | *Ascaris suum* | Eukaryota | 2.81 | 4 | | | Oxidoreductases | protein-protein | multimer* | ['Asymmetry: C1', ‘BA1: Hetero 4-mer – ABCD’, ‘BA2: Hetero 4-mer – ABCD’] | C:34-66; D:28-54 |
| *4A01* | AB | alpha | H^+^-translocating M-PPase | *Vigna radiata* | Eukaryota | 2.35 | 1 | | | Membrane-Integral Pyrophosphatases (M-PPases) | protein-ligand | m-m | ['Asymmetry: A2', 'BA1: Homo 2-mer - A2'] | A:42-66; B:42-66 |
| *4AV3* | AB | alpha | Na^+^ -translocating M-PPase with metal ions in active site | *Thermotoga maritima* | Bacteria | 2.60 | 1 | | | Membrane-Integral Pyrophosphatases (M-PPases) | protein-protein | m-m | ['Asymmetry: A2', 'BA1: Homo 2-mer - A2'] | A:695-726; B:2-113,115-211,221-590,599-694 |
| *4COF* | AB | alpha | GABAR receptor (3 homopentamer) | *Homo sapiens* | Eukaryota | 2.97 | 1 | | | Cys-Loop Receptor Family | protein-protein | multimer | ['Asymmetry: A5', 'BA1: Homo 5-mer - A5'] | A:1-214; B:10-215 |
| *4CZB* | AB | alpha | NhaP1 Na^+^/H^+^ antiporter, pH 8 | *Methanocaldococcus jannaschii* | Archaea | 3.50 | 2 | | | Antiporters | protein-protein | multimer | ['Asymmetry: A4', 'BA1: Homo 2-mer - A2', 'BA2: A2'] |  |
| *4DJH* | AB | alpha | Κ - opioid receptor in complex with JDTic | *Homo sapiens* | Eukaryota | 2.90 | 1 | | | G Protein-Coupled Receptors (GPCRs) | protein-ligand | m-m* | ['Asymmetry: A2', 'BA1: Homo 2-mer - A2', 'BA2: A2'] | A:1002-1161; B:55-261,1002-1161 |
| *4EV6* | AB | alpha | CorA Mg^2+^Transporter | *Methanocaldococcus jannaschii* | Archaea | 3.20 | 1 | | | CorA Superfamily Ion Transporters | protein-protein | multimer | ['Asymmetry: A5', 'BA1: Homo 5-mer - A5'] | A:3-248; B:4-251 |
| *4EZC* | AB | alpha | UT-B Urea Transporter | *Bos taurus* | Eukaryota | 2.36 | 1 | | | Channels: Urea Transporters | protein-ligand | multimer | ['Asymmetry: A3', 'BA1: Homo 3-mer - A3'] |  |
| *4F4L* | AC | alpha | Voltage-Gated Sodium Channel (Na) | *Magnetococcus marinus* | Bacteria | 3.49 | 1 | | | Channels: Potassium, Sodium, & Proton Ion-Selective | protein-protein | multimer | ['Asymmetry: A4', 'BA1: Homo 4-mer - A4'] |  |
| *4F4S* | AB | alpha | Structure of the yeast F1Fo ATPase c10 ring with bound oligomycin | *Saccharomyces cerevisiae* (strain ATCC 204508 / S288c) | Bacteria | 1.90 | 10 | | | MEMBRANE PROTEIN/ANTIBIOTIC | protein-protein | multimer | ['Asymmetry: Homo 10-mer - A10 ', 'BA1: Homo 10-mer - A10', 'BA2: Homo 10-mer - A10'] |  |
| *4G1U* | AB | alpha | HmuUV heme transporter | *Yersinia pestis* | Bacteria | 3.00 | 2 | | | ATP Binding Cassette (ABC) Transporters | protein-protein | multimer* | ['Asymmetry: A2B2', 'BA1: Hetero 4-mer - A2B2'] | A:298-326; B:4-28,48-106,114-297 |
| *4GX0* | AB | alpha | GsuK multi-ligand gated K^+^ channel, L97D mutant | *Geobacter sulfurreducens* | Bacteria | 2.60 | 1 | | | Channels: Potassium, Sodium, & Proton Ion-Selective | protein-protein | multimer | ['Asymmetry: Homo 4-mer - A4', 'BA1: A4'] | A:121-261,350-480; B:120-564 |
| *4HKR* | AB | alpha | Orai Calcium release-activated calcium (CRAC) channel | *Drosophila melanogaster* | Eukaryota | 3.35 | 1 | | | Channels: Calcium Ion-Selective | protein-protein | m-m | ['Asymmetry: Homo 6-mer - A6'] | A:181-190,220-235; B:181-190,220-235 |
| *4HYG* | AB | alpha | PSH presenilin/SPP homologue aspartate protease (C222 space group) | *Methanoculleus marisnigri* | Archaea | 3.32 | 1 | | | Intramembrane Proteases | protein-protein | multimer | ['Asymmetry: Homo 4-mer - A4', 'BA1: A4'] | A:38-40,180-211,235-243; B:38-40,177-209,235-238 |
| *4J72* | AB | alpha | MraY phospho-MurNAc-pentapeptide translocase | *Aquifex aeolicus* | Bacteria | 3.30 | 1 | | | PNPT Superfamily | protein-protein | m-m | ['Asymmetry: A2', 'BA1: Homo 2-mer - A2'] | A:49-69; B:49-69,312-333,122-125 |
| *4J7C* | IJ | alpha | KtrAB potassium ion transporter | *Bacillus subtilis* | Bacteria | 3.50 | 2 | | | Superfamily of K Transporters (SKT proteins) | protein-protein | multimer* | ['Asymmetry: A8B4', 'BA1: Hetero 10-mer - A8B2', 'BA2: A8B2', 'BA3: A8B4'] | I:103-104; J:103-104 |
| *4JKV* | AB | alpha | Smoothened (SMO) receptor with bound antagonist, LY2940680 | *Homo sapiens* | Eukaryota | 2.45 | 1 | | | G Protein-Coupled Receptors (GPCRs) | protein-ligand | m-m* | ['Asymmetry: A2', 'BA1: Homo 2-mer - A2'] | A:-1-106,190-221; B:0-106,190-209 |
| *4JQ6* | AB, AC | alpha | Proteorhodopsin (blue-light absorbing), BPR | uncultured bacterium | Bacteria | 2.31 | 1 | | | Bacterial and Algal Rhodopsins | protein-protein | multimer | ['Asymmetry: Homo 6-mer - A6'] | A:97-104,151-168; B:98-103,152-162; C:97-104,150-164 |
| *4KLY* | AB, BC | alpha | Proteorhodopsin (blue-light absorbing); BPR, D97N mutant | gamma proteobacterium | Bacteria | 2.70 | 1 | | | Bacterial and Algal Rhodopsins | protein-protein | multimer | ['Asymmetry: A5', 'BA1: Homo 5-mer - A5'] | A:173-176,213-213; B:212-214; C:212-214 |
| *4MBS* | AB | alpha | CCR5 chemokine receptor with bound Maraviroc | *Homo sapiens* | Eukaryota | 2.71 | 1 | | | G Protein-Coupled Receptors (GPCRs) | protein-ligand | m-m | ['Asymmetry: A2', 'BA1: Monomer', 'BA2: A'] | A:1001-1054; B:1001-1054 |
| *4MRN* | AB | alpha | Atm1-type ABC exporter, apo protein | *Novosphingobium aromaticivorans* | Bacteria | 2.50 | 1 | | | ATP Binding Cassette (ABC) Transporters | protein-protein | m-m | ['Asymmetry: A2', 'BA1: Homo 2-mer - A2'] | A:344-539,541-591,593-607; B:344-539,541-591,593-606 |
| *4MT4* | AB | beta | CmeC bacterial multi-drug efflux transporter outer membrane channel | *Campylobacter jejuni* | Bacteria | 2.37 | 1 | | | Beta-Barrel Membrane Proteins: Monomeric/Dimeric | protein-protein | multimer | ['BA1: Homo 3-mer - A3', 'Asymmetry: A3'] | A:1-81,129-296,338-472; B:1-82,127-295,337-473 |
| *4MYC* | AB | alpha | Atm1 mitochondrial ABC transporter, apo form | *Saccharomyces cerevisiae* | Eukaryota | 3.06 | 1 | | | ATP Binding Cassette (ABC) Transporters | protein-protein | multimer | ['Asymmetry: Homo 2-mer - A2', 'BA1: A2'] | A:186-224,282-333,397-695; B:181-229,283-332,396-695 |
| *4O6M* | AB | alpha | AF2299 CDP-alcohol phosphotransferase w. bound CMP | *Archaeoglobus fulgidus* | Archaea | 1.90 | 1 | | | CDP-Alcohol Phosphotransferases | protein-ligand | m-m | ['Asymmetry: A2', 'BA1: Homo 2-mer - A2'] | A:1-133; B:1-135 |
| *4O6Y* | AB | alpha | Cytochrome b | *Arabidopsis thaliana* | Eukaryota | 1.70 | 1 | | | Oxidoreductases | protein-ligand | m-m | ['Asymmetry: A2', 'BA1: Homo 2-mer - A2'] |  |
| *4P6V* | BD, BE, CD, CF, DE, EF | alpha | Na^+^ -pumping NADH:quinone oxidoreductase (Na^+^ -NQR) | *Vibrio cholerae* | Bacteria | 3.50 | 6 | | | Oxidoreductases | protein-protein | multimer | ['Asymmetry: ABCDEF', 'BA1: Hetero 6-mer - ABCDEF'] | A:376-446; B:38-112,119-250,267-326,329-332; C:31-256; F:30-408 |
| *4PHZ* | AB, BC | alpha | Crystal structure of particulate methane monooxygenase from Methylocystis sp. ATCC 49242 (Rockwell) | *Methylococcus capsulatus* | Bacteria | 2.59 | 12 | | | Oxidoreductases | protein-protein | multimer* | ['Asymmetry: Hetero 11-mer - A3B3C3D2', 'BA1: Hetero 11-mer - A3B3C3D2 '] | A:29-157,264-416  C:199-225 |
| *4PIR* | AB | alpha | Serotonin 5-HT receptor | Mus musculus | Eukaryota | 3.50 | 2 | | | Cys-Loop Receptor Family | protein-protein | multimer | ['Asymmetry: A5B5', 'BA1: Hetero 10-mer - A5B5'] | A:8-218,312-334,399-428; B:8-217,309-334,399-423 |
| *4PL0* | AB | alpha | McjD antimicrobial peptide transporter | *Escherichia coli* | Bacteria | 2.70 | 1 | | | ATP Binding Cassette (ABC) Transporters | protein-ligand | m-m | ['Asymmetry: A2', 'BA1: Homo 2-mer - A2'] | A:97-131,193-245,317-579; B:97-131,193-245,318-579 |
| *4QNC* | AB | alpha | semiSWEET transporter in occluded state | *Leptospira biflexa* | Bacteria | 2.39 | 1 | | | SWEET and semiSWEET Transporters, and Their Relatives | protein-protein | m-m | ['Asymmetry: A2', 'BA1: Homo 2-mer - A2'] |  |
| *4QTN* | AB | alpha | PnuC vitamin B transporter | *Neisseria mucosa* | Bacteria | 2.80 | 1 | | | SWEET and semiSWEET Transporters, and Their Relatives | protein-ligand | multimer | ['Asymmetry: A3', 'BA1: Homo 3-mer - A3'] |  |
| *4R0C* | AB | alpha | YdaH transporter | *Alcanivorax borkumensis* | Bacteria | 2.96 | 1 | | | AbgT Family of Transporters | protein-protein | multimer | ['Asymmetry: A4', 'BA1: Homo 4-mer - A4'] | B:112-117 |
| *4RDQ* | AB | alpha | Bestrophin-1 (BEST1) Ca^2+^ -activated Cl^-^channel | *Gallus gallus* | Eukaryota | 2.85 | 5 | | | Channels: Other Ion Channels | protein-antibody | multimer* | ['Asymmetry: A5B5C5', 'BA1: Hetero 15-mer - A10B5'] | A:95-230,306-367; B:95-230,307-367 |
| *4RI2* | AB | alpha | PsbS photoprotection protein | *Spinacia oleracea* | Eukaryota | 2.35 | 1 | | | Photoprotection Proteins | protein-ligand | m-m | ['Asymmetry: A2', 'BA1: Homo 2-mer - A2'] | A:108-133; B:107-133 |
| *4RNG* | AC | alpha | semiSWEET transporter in occluded state | *Thermodesulfovibrio yellowstonii* | Bacteria | 2.40 | 1 | | | SWEET and semiSWEET Transporters, and Their Relatives | protein-protein | multimer | ['Asymmetry: Homo 2-mer - A2', 'BA1: A2', 'BA2: A2', 'BA3: A6'] |  |
| *4RY2* | AB | alpha | Peptidase-containing ABC transporter (PCAT) | *Ruminiclostridium thermocellum* | Bacteria | 3.61 | 1 | | | ATP Binding Cassette (ABC) Transporters | protein-protein | m-m | ['Asymmetry: A2', 'BA1: Homo 2-mer - A2'] | A:12-165,233-283,332-384,450-471,484-724; B:12-165,235-280,331-387,448-471,484-722 |
| *4TQU* | MN | alpha | Alginate transporter AlgM1M2SS with bound periplasmic protein AlgQ2 | *Sphingomonas sp.* | Bacteria | 3.20 | 4 | | | ATP Binding Cassette (ABC) Transporters | protein-protein | multimer* | ['Asymmetry: A2BCD', 'BA1: Hetero 5-mer - A2BCD'] | M:64-75 |
| *4UC1* | AB | alpha | Translocator protein (TSPO), A139T SeMet1 C121 | *Rhodobacter sphaeroides* | Bacteria | 1.80 | 1 | | | Translocator Protein (18 kDA) TSPO | protein-ligand | multimer | ['Asymmetry: Homo 2-mer - A2', 'BA1: A2'] |  |
| *4UV3* | AB | beta | CsgG bacterial amyloid secretion channel | *Escherichia coli* | Bacteria | 3.59 | 1 | | | Beta-Barrel Membrane Proteins: Monomeric/Dimeric | protein-protein | multimer | ['BA1: Homo 9-mer - A9', 'BA2:A9', 'Asymmetry: A18'] | A:2-130,156-178,211-257; B:2-131,158-180,212-257 |
| *4WD7* | AB | alpha | KpBest Bestrophin homolog of the BEST1 Ca^2+^ -activated Cl^-^ channel (&Delta;C7) | *Klebsiella pneumoniae* | Bacteria | 2.90 | 1 | | | Channels: Other Ion Channels | protein-protein | multimer | ['Asymmetry: A5', 'BA1: Homo 5-mer - A5'] | A:79-206,262-289; B:80-207,262-291 |
| *4WFE* | AB | alpha | Human TRAAK K+ channel in a K+ bound conductive conformation | *Homo sapiens* | Eukaryota | 2.50 | 6 | | | Metal transporter | protein-antibody | multimer* | ['Asymmetry: Hetero 6-mer - A2B2C2', 'BA1: Hetero 6-mer - A2B2C2'] | A:104-109; B:106-109 |
| *4WGV* | AC | alpha | SLC11 (NRAMP) transition-metal ion transporter in complex with nanobodies | *Staphylococcus capitis* | Bacteria | 3.10 | 2 | | | Solute Carrier (SLC) Transporter Superfamily | protein-antibody | multimer* | ['Asymmetry: A2B2', 'BA1: Hetero 4-mer - A2B2'] | A:396-439; B:2-121 |
| *4WIS* | AB | alpha | TMEM16 Ca^2+^ -activated lipid scramblase, crystal form 1 | *Nectria haematococca* | Eukaryota | 3.30 | 1 | | | TMEM16 Family Proteins | protein-protein | m-m | ['Asymmetry: A2', 'BA1: Homo 2-mer - A2'] | A:19-129,141-154,628-656,660-684,692-719; B:19-129,141-151,629-656,660-684,692-719 |
| *4X5M* | BC | alpha | semiSWEET transporter in inward-open conformation (crystal I) | *Escherichia coli* | Bacteria | 2.00 | 1 | | | SWEET and semiSWEET Transporters, and Their Relatives | protein-protein | multimer | ['Asymmetry: Homo 2-mer - A2', 'BA1: A2'] |  |
| *4XYD* | AB | alpha | Nitric Oxide Reductase BC complex | *Roseobacter denitrificans* | Bacteria | 2.85 | 2 | | | Nitric Oxide Reductases | protein-ligand | m-m* | ['Asymmetry: AB', 'BA1: Hetero 2-mer - AB'] |  |
| *4YMS* | CD | alpha | Art(QN) amino acid importer | *Caldanaerobacter tengcongensis* | Bacteria | 2.80 | 2 | | | ATP Binding Cassette (ABC) Transporters | protein-protein | multimer* | ['Asymmetry: A2B2', 'BA1: Hetero 4-mer - A2B2'] |  |
| *4YZF* | AB | alpha | Erythrocyte Band 3 anion exchanger | *Homo sapiens* | Eukaryota | 3.50 | 3 | | | Solute Carrier Family 4 (anion exchanger) | protein-antibody | multimer* | ['Asymmetry: A4B4C4', 'BA1: Hetero 6-mer - A4B2', 'BA2: A2B2C2'] | A:554-566,641-648,742-752; B:554-566,641-648,742-752 |
| *5A1S* | AB | alpha | CitS Citrate symporter | *Salmonella enterica* | Bacteria | 2.50 | 1 | | | Solute Carrier (SLC) Transporter Superfamily | protein-peptide | multimer* | ['Asymmetry: A4', 'BA1: Homo 2-mer - A2', 'BA2: A2'] | B:250-256 |
| *5A2N* | AB | alpha | NRT1.1 nitrate transporter, apo form | *Arabidopsis thaliana* | Eukaryota | 3.70 | 1 | | | Major Facilitator Superfamily (MFS) Transporters | protein-protein | m-m | ['Asymmetry: A2', 'BA1: Homo 2-mer - A2'] | A:124-141,270-325,452-460; B:124-141,270-325,452-460 |
| *5AEX* | AB | alpha | Mep2 ammonium transceptor | *Saccharomyces cerevisiae* | Eukaryota | 3.20 | 1 | | | Channels: Amt/Mep/Rh proteins | protein-protein | multimer | ['Asymmetry: Homo 3-mer - A3', 'BA1: A3', 'BA2: A3'] |  |
| *5AWW* | AB, AC | alpha | Precise Resting State of Thermus thermophilus SecYEG | *Thermus thermophilus* (strain HB8 / ATCC 27634 / DSM 579) | Bacteria | 2.72 | 3 | | | PROTEIN TRANSPORT/IMMUNE SYSTEM | protein-protein | multimer | ['Asymmetry: Hetero 3-mer - ABC', 'BA1: Hetero 3-mer - ABC'] |  |
| *5AZS* | AB | beta | OprJ drug discharge outer membrane protein | *Pseudomonas aeruginosa* | Bacteria | 3.10 | 1 | | | Beta-Barrel Membrane Proteins: Monomeric/Dimeric | protein-protein | multimer | ['BA1: Homo 3-mer - A3', 'Asymmetry: A3'] | A:1-19,33-80,124-292,333-443; B:1-21,30-80,125-292,332-443 |
| *5B57* | AB | alpha | BhuU/BhuV haem importer, inward facing | *Burkholderia cenocepacia* | Bacteria | 2.80 | 2 | | | ATP Binding Cassette (ABC) Transporters | protein-protein | multimer* | ['Asymmetry: A2B2', 'BA1: Hetero 4-mer - A2B2'] | A:135-140; B:134-139 |
| *5BUN* | AB | beta | ST50 discharge outer membrane protein | *Salmonella enterica* | Bacteria | 2.98 | 1 | | | Beta-Barrel Membrane Proteins: Monomeric/Dimeric | protein-protein | multimer | ['BA1: Homo 3-mer - A3', 'Asymmetry: A3'] | A:1-39,78-244,293-427; B:1-36,78-243,289-425 |
| *5C78* | AD | alpha | PglK lipid-linked oligosaccharide flippase, apo-inward structure 1 | *Campylobacter jejuni* | Bacteria | 2.90 | 1 | | | ATP Binding Cassette (ABC) Transporters | protein-protein | multimer | ['Asymmetry: A4', 'BA1: Homo 2-mer - A2', 'BA2: A2'] | A:1-18,98-150,194-255,313-564; D:1-15,100-147,149,197-253,315-564 |
| *5C8J* | IL | alpha | DUF106 YidC-like protein | *Methanocaldococcus jannaschi* | Archaea | 3.50 | 5 | | | Sec and Translocase Proteins | protein-antibody | multimer* | ['Asymmetry: C1', ‘BA1: Hetero 3-mer – ABC’, ‘BA2: Hetero 3-mer – ABC’, ‘BA3: Hetero 3-mer – ABC’, ‘BA4: Hetero 3-mer – ABC’] | I:46-101; L:45-97 |
| *5CFB* | AB | alpha | Human glycine receptor (hGlyR-α; 3) in complex with strychnine | *Homo sapiens* | Eukaryota | 3.04 | 1 | | | Cys-Loop Receptor Family | protein-protein | multimer | ['Asymmetry: A5', 'BA1: Homo 5-mer - A5'] | A:9-214; B:9-215 |
| *5CTG* | AB | alpha | SWEET transporter in a homotrimeric complex | *Oryza sativa* | Eukaryota | 3.10 | 1 | | | SWEET and semiSWEET Transporters, and Their Relatives | protein-protein | multimer | ['Asymmetry: A3', 'BA1: Homo 3-mer - A3'] |  |
| *5DO7* | AB | alpha | ABCG5/ABCG8 sterol transporter | *Homo sapiens* | Eukaryota | 3.93 | 2 | | | ATP Binding Cassette (ABC) Transporters | protein-protein | multimer* | ['Asymmetry: A2B2', 'BA1: Hetero 2-mer - AB', 'BA2: AB'] | A:34-46,66-362; B:23-44,87-321,342-366,394-410 |
| *5EKP* | AB | alpha | GtrB polyisoprenyl-glycosyltransferase (PI-GT) | *Synechocystis* sp. PCC6803 | Bacteria | 3.19 | 1 | | | Glycosyltransfereases | protein-protein | multimer | ['Asymmetry: A4', 'BA1: Homo 4-mer - A4'] | A:-3-127,146-197,298-313; B:-3-124,133-197,299-316 |
| *5EUL* | EY | alpha | SecYE translocon in complex with SecA | *Geobacillus thermodenitrificans* | Bacteria | 3.70 | 4 | | | Sec and Translocase Proteins | protein-protein | multimer* | ['Asymmetry: ABCD', 'BA1: Hetero 4-mer - ABCD'] | Y:145-145,201-213,245-258,268-272,296-300 |
| *5H3O* | AB | alpha | Cyclic-nucleotide-gated (CNG) channel | *Caenorhabditis elegans* | Eukaryota | 3.50 | 1 | | | Channels: Potassium, Sodium, & Proton Ion-Selective | protein-ligand | multimer | ['Asymmetry: A4', 'BA1: Homo 4-mer - A4'] | A:422-620; B:419-620 |
| *5HK7* | AB | alpha | Bacterial sodium channel pore | *Alkalilimnicola ehrlichii* (strain ATCC BAA-1101 / DSM 17681 / MLHE-1) | Bacteria | 2.95 | 4 | | | Transport protein | protein-protein | multimer | ['Asymmetry: C4', 'BA1: Homo 4-mer - A4'] | A:250-286; B:250-285 |
| *5J4I* | AB | alpha | Crystal Structure of the L-arginine/agmatine antiporter from E. coli at 2.2 Angstroem resolution | *Escherichia coli* | Bacteria | 2.21 | 2 | | | Transport protein | protein-protein | m-m | ['Asymmetry: Homo 2-mer - A2', 'BA1: Homo 2-mer - A2'] |  |
| *5KBN* | AB | alpha | Fluc F^-^ ion channel homolog in complex Ec2-S9 monobody F801 mutant | *Escherichia coli* | Bacteria | 2.48 | 2 | | | Channels: Fluc Family | protein-protein | multimer* | ['Asymmetry: C1', 'BA1: Hetero 2-mer - AB', 'BA2: Hetero 2-mer - AB'] |  |
| *5KHN* | AB | alpha | HpnN hopanoid transporter (crystal form I) | *Burkholderia multivorans* | Bacteria | 3.44 | 1 | | | Multi-Drug Efflux Transporters | protein-protein | m-m | ['Asymmetry: A2', 'BA1: Homo 2-mer - A2'] | A:42-226,234-273,479-715: B:40-226,233-272,476-715 |
| *5KXI* | AB, AE | alpha | Nicotinic Acetylcholine α4β2 Receptor | *Homo sapiens* | Eukaryota | 3.94 | 2 | | | Cys-Loop Receptor Family | protein-ligand | multimer | ['Asymmetry: C1', 'BA1: Hetero 5-mer - A3B2’] | A:8-215; B:1-207; E:1-207 |
| *5L22* | AB | alpha | PrtD Type-1 secretion system ABC transporter | *Aquifex aeolicus* | Bacteria | 3.15 | 1 | | | ATP Binding Cassette (ABC) Transporters | protein-ligand | m-m | ['Asymmetry: A2', 'BA1: Homo 2-mer - A2'] | A:11-19,84-131,178-236,301-315,326-559; B:10-20,85-131,177-239,295-315,326-559 |
| *5MKK* | AB | alpha | TmrAB antigen transporter homolog | *Thermus thermophilus* | Bacteria | 2.70 | 2 | | | ATP Binding Cassette (ABC) Transporters | protein-protein | m-m* | ['Asymmetry: C1', 'BA1: Hetero 2-mer - AB'] | A:104-145,206-247,314-603; B:84-131,191-230,310-578 |
| *5MRW* | AB, AC, BD | alpha | Potassium-importing KdpFABC membrane complex | *Escherichia coli* | Bacteria | 2.90 | 12 | | | Superfamily of K Transporters (SKT proteins) | protein-protein | multimer* | ['Asymmetry: A3B3C3D3', 'BA1: Hetero 4-mer - ABCD', 'BA2: ABCD', 'BA3: ABCD'] | B:90-161,163-199,278-568; C:41-190 |
| *5N77* | AB | alpha | CorA Mg^2+^ Transporter cytoplasmic domain with bound Mg^2+^ | *Escherichia coli* | Bacteria | 2.80 | 1 | | | CorA Superfamily Ion Transporters | protein-protein | multimer | ['Asymmetry: A5', 'BA1: Homo 5-mer - A5'] |  |
| *5NKQ* | AB | alpha | Crystal structure of a dual topology fluoride ion channel. | *Bordetella pertussis* (strain Tohama I / ATCC BAA-589 / NCTC 13251) | Bacteria | 2.17 | 4 | | | Transport protein | protein-protein | multimer | ['Asymmetry: Hetero 8-mer - A4B4', 'BA1: Hetero 4-mer - A2B2', 'BA2: Hetero 4-mer - A2B2'] |  |
| *5SV0* | AB | alpha | ExbB/ExbD complex associated with TonB complex, pH 7.0 | *Escherichia coli* | Bacteria | 2.60 | 1 | | | Channels: Other Ion Channels | protein-protein | multimer | ['Asymmetry: A10', 'BA1: Homo 5-mer - A5', 'BA2: A5'] | A:51-107,109-126,203-203,205-234; B:51-107,109-126,202-234 |
| *5SY1* | AB | alpha | STRA6 retinol-uptake receptor in complex with calmodulin (CaM) | *Danio rerio* | Eukaryota | 3.90 | 2 | | | Novel Receptors | protein-protein | multimer | ['Asymmetry: A2B2', 'BA1: Hetero 4-mer - A2B2'] | A:452-574,598-630; B:443-574,598-630 |
| *5T0O* | AB | alpha | CmeB multi-drug efflux transporter, C2 space group | *Campylobacter jejuni* | Bacteria | 3.15 | 1 | | | Multi-Drug Efflux Transporters | protein-protein | multimer | ['Asymmetry: A3', 'BA1: Homo 3-mer - A3'] | A:438-1033; B:1-222,230-437,873-1033 |
| *5TIN* | AB | alpha | Crystal Structure of Human Glycine Receptor alpha-3 Mutant N38Q Bound to AM-3607 | *Homo sapiens* | Eukaryota | 2.61 | 5 | | | Transport protein | protein-protein | multimer | ['Asymmetry: Homo 5-mer - A5', 'BA1: Homo 5-mer - A5'] | A:8-220; B:6-218 |
| *5TQQ* | AB | alpha | CLC-K chloride ion channel, class 1 | *Bos taurus* | Eukaryota | 3.76 | 5 | | | Channels: Other Ion Channels | protein-antibody | multimer* | ['Asymmetry: A2B2C2', 'BA1: Hetero 6-mer - A4B2'] | A:535-605,618-683; B:540-605,618-683 |
| *5U1D* | AB | alpha | Transporter associated with antigen processing (TAP) bound to ICP47 | *Homo sapiens* | Eukaryota | 4.00 | 3 | | | ATP Binding Cassette (ABC) Transporters | protein-peptide | both | ['Asymmetry: ABC', 'BA1: Hetero 3-mer - ABC'] | A:256-296,355-402,470-484,492-742; B:218-262,322-362,438-681 |
| *5U6O* | AB | alpha | HCN1 hyperpolarization-activated channel | *Homo sapiens* | Eukaryota | 3.50 | 1 | | | Channels: Potassium, Sodium, & Proton Ion-Selective | protein-protein | multimer | ['Asymmetry: A4', 'BA1: Homo 4-mer - A4'] | A:403-586; B:402-586 |
| *5UNI* | AB | alpha | Critical role of water molecules for proton translocation of the membrane-bound transhydrogenase | *Thermus thermophilus* (strain HB27 / ATCC BAA-163 / DSM 7039) | Bacteria | 2.20 | 2 | | | Oxidoreductases | protein-protein | m-m | ['Asymmetry: Hetero 2-mer - AB', 'BA1: Hetero 2-mer - AB', 'BA2: Hetero 4-mer - A2B2'] |  |
| *5V6P* | AB | alpha | ER-associated protein degradation (ERAD) protein. Hrd1 channel in complex with Hrd3 | *Saccharomyces cerevisiae* | Eukaryota | 4.10 | 1 | | | Sec and Translocase Proteins | protein-protein | m-m | ['Asymmetry: A2', 'BA1: Homo 2-mer - A2'] | A:221-265; B:221-265 |
| *5VRE* | AC | alpha | TMEM175 lysosomal K^+^ channel | *Chamaesiphon minutus* | Eukaryota | 3.30 | 1 | | | Channels: Potassium, Sodium, & Proton Ion-Selective | protein-protein | multimer | ['Asymmetry: A4', 'BA1: Homo 4-mer - A4'] | A:145-153; B:145-153 |
| *6BAA* | AB | alpha | Cryo-EM structure of the pancreatic beta-cell KATP channel bound to ATP and glibenclamide | *Rattus norvegicus* | Eukaryota | 3.63 | 8 | | | Metal transporter | protein-protein | multimer | ['Asymmetry: Hetero 8-mer - A4B4', 'BA1: Hetero 8-mer - A4B4'] | A:32-65,174-352; B:32-66,176-352 |
| *6F0U* | AB | alpha | GLIC mutant E35A | *Gloeobacter violaceus* (strain PCC 7421) | Bacteria | 2.35 | 5 | | | Membrane protein | protein-protein | multimer | ['Asymmetry: Homo 5-mer - A5', 'BA1: Homo 5-mer - A5'] | A:5-191; B:5-191 |
| *7AHL* | AB | beta | α- hemolysin | *Staphylococcus aureus* | Bacteria | 1.90 | 1 | | | Adventitious Membrane Proteins: Beta-sheet Pore-forming Toxins/Attack Complexes | protein-protein | multimer | ['Asymmetry: A7', 'BA1: Homo 7-mer - A7'] | A:1-106,150-293; B:1-108,153-293 |
